# The Role of Stratified Cumulative Antibiograms in the (Choice of Appropriate Antibiotics in Urinary Tract Infection) Management of Urinary Tract Infections

**DOI:** 10.3390/pathogens14020141

**Published:** 2025-02-03

**Authors:** Vaclava Adamkova, Michaela Matouskova, Vanda Gabriela Adamkova, Michal Huptych, Marcela Fontana

**Affiliations:** 1 Clinical Microbiology and ATB Centre, General University Hospital, 128 08 Prague, Czech Republic; 2 Urocentrum Praha, 120 00 Prague, Czech Republic; matouskova@urocentrum.cz (M.M.); cechovamarcela@seznam.cz (M.F.); 3 Department of Plant Sciences, University of Cambridge, Cambridge CB2 3EA, UK; 4 Czech Institute of Informatics, Robotics and Cybernetics (CIIRC), Czech Technical University in Prague, 160 00 Prague, Czech Republic; michalhuptych@seznam.cz; 5 Department of Urology, University Hospital Motol, 2nd Faculty of Medicine, Charles University, 150 06 Prague, Czech Republic

**Keywords:** urinary tract infections, stratified cumulative antibiograms, resistance, *Escherichia coli*, antibiotics+

## Abstract

Urinary tract infections (UTIs) are one of the most common bacterial diseases both in communities and in hospitalized patients, and at the same, time they are one of the most common indications for the use of antibiotics. UTI guidelines are generally available nationally or internationally, but they do not address all aspects of UTI treatment for different patient cohorts, age, gender, or comorbidities. The aim of the study was to point out the importance of stratified cumulative antibiograms at the level of individual health care facilities and the significant differences between epidemiological data, not only at the national level, but also at the local level. Our study analyses data from 383 patients with UTIs from a hospital department, General University Hospital (GUH), and 272 patients from an outpatient medical facility, Urocentrum (UC). This analysis focuses on the most common UTI causative agent, *Escherichia coli*, its representation as the causative agent of UTI in patients with complicated acute cystitis (N30), and its representation in complicated acute cystitis in patients with prostate cancer (C61). In addition to the frequency of occurrence, a sub-analysis of the incidence of resistance of *E. coli* to commonly used antibiotics by age, gender, diagnosis, and medical facility was performed. Results: The most common causative agent of UTI was *E. coli*. In patients with N30, it was 70% in GUH and 54% in UC, but in oncological patients with UTI, it was only 39% and 35%, respectively. In patients with UTI in C61, there was a significant difference in susceptibility of *E. coli* between individual health care facilities. Lower resistance was found in UC opposite to GUH isolates in ampicillin, with 29.8% vs. 65%, *p* = 0.001; amoxicillin/clavulanic acid, with 8.5% vs. 30%, *p* = 0.01; with 2.1% vs. 17.5% in pivmecillinam, *p* = 0.01; with 10.6% vs. 37.5% in co-trimoxazole, *p* = 0.003; and ciprofloxacin, with 10.6% vs. 30%, *p* = 0.04. The study shows significant differences in the sensitivity of urinary *E. coli* isolates in patients in relation to age, gender, medical devices, and the presence of comorbidities.

## 1. Introduction

Urinary tract infection (UTI) is one of the most common bacterial diseases occurring in all age groups [1]. UTI affects both sexes but is more common in women. It is reported that 50–60% of women will experience at least one episode of UTI during their lifetime [2]. Worldwide, the incidence of UTIs is estimated at 150 million cases per year. According to data collected from the US National Ambulatory Medical Care Survey and the National Hospital Ambulatory Medical Care Survey, UTIs( led to approximately seven million doctor’s office visits and one million emergency room visits, resulting in one hundred thousand hospitalizations. UTI consultations account for 6% of all medical visits, which accounts for approximately seven million visits per year in the US [3,4].

The majority of UTIs (95%) are of bacterial origin, and *Escherichia coli* accounts for up to 90% of findings, depending on clinical conditions. UTIs can also be induced by other Gram-negative rods or Gram-positive cocci, but to a much lesser extent [5]. From a clinical point of view, it is important to divide UTIs into uncomplicated and complicated. Uncomplicated urinary tract infection is understood to be a UTI in non-gravid, premenopausal women without serious comorbidities and without structural or functional abnormalities of the urogenital tract. Complicated UTIs arise in individuals predisposed to infection by anatomical or functional urinary tract abnormalities, the presence of a foreign body (e.g., permanent urinary catheter, lithiasis and others), reduced immunity, or concomitant comorbidities. Since microbiological urine testing in patients with uncomplicated lower urinary tract infections is not routinely recommended and required, treatment of these infections is often initiated empirically according to national guidelines, where available. Depending on the type, UTIs are commonly treated with antibiotics such as trimethoprim and sulfamethoxazole, β-lactams, fluoroquinolones, nitrofurantoin and phosphomycin. These antibiotics are preferred because of their broad spectrums of efficacy, good tolerability, and beneficial pharmacokinetic properties [6]. However, this approach often leads to an inappropriate choice of antibiotic, and thus contributes to an increase in bacterial resistance, including to multidrug-resistant (MDR) strains of bacterial pathogens. At the same time, the occurrence of bacteria resistant to oral forms of antibiotics is increasing, which significantly complicates therapeutic options for the treatment of uncomplicated UTIs in outpatient modes [7]. Global antibiotic resistance of pathogenic bacteria is increasing every day. However, the resistance differs at the individual level, both locally and internationally [8,9,10]. Properly administered antibiotics to treat urinary tract infections shorten hospital stays, improve patient outcomes, and reduce health care costs [11].

To maintain the effectiveness of existing antibiotics and the related possibility of effective treatment, active cooperation between clinicians and microbiologists in monitoring and predicting the occurrence of resistance is essential. Systematic surveillance makes it possible to monitor long-term trends in the development of resistance and to evaluate the impact of intervention measures aimed at reducing the occurrence and spread of resistant bacteria [12]. In 2015, the World Health Organization prepared a global action plan, where antimicrobial resistance surveillance is mentioned as a cornerstone of antimicrobial resistance control [13].

Adequate and rational antibiotic therapy therefore requires the following [14]:

(1) Sensitive and specific laboratory diagnostics to confirm the infectious etiology of the condition (biomarkers of infection);
(2) Timely and correctly performed microbiological examination for early identification of the causative agent (or molecular diagnosis of the pathogen);
(3) Appropriate empirical antibiotic treatment (broad-spectrum antibiotics and/or their combination, early administration, adequate dosing, proportional duration of administration);
(4) The identification of patient risk factors associated with a higher incidence of infections caused by MDR strains.

For these reasons, other factors, including the local level of resistance of the most isolated causative agents, are increasingly included in empirical antimicrobial therapy, which should lead to the most effective treatment initiated.

Antibiotic stewardship should be seen as a strategy within everyday activities. The word strategy comes from the Greek word “strategos”, which means a general but unified set of maneuvers leading to the destruction of the enemy. The key word here is general, rather than specific, because only the fulfilment of the strategy is a specific activity, which is carried out at the local, not national, level [15].

A cumulative antibiogram can be defined as an overview of the incidence of resistance to individual antibiotics in bacteria isolated from the patient’s biological material [16]. The usefulness of cumulative antibiograms depends primarily on robust input data (i.e., standardized testing of bacterial susceptibility to antibiotics) as well as transparency and consistent production of antibiograms across different microbiology laboratories [17]. Reliable cumulative antibiograms can significantly help, both in preventing incorrect antibiotic selection, and in reducing the excessive use of broad-spectrum antibiotics at a time when the results of microbiological examination and antibiotic sensitivity determination are not yet available for a particular patient [18].

Data stratification is important for defining antimicrobial stewardship (AMS) problems. If the laboratory is conducting multi-subject examinations with different patient compositions, it is necessary to analyze, at least, data obtained from patients in different age groups and with different diagnoses. Furthermore, it is suitable and important for the purposes of AMS to process isolates of one species of bacteria from various clinically valid materials for individual facilities or clinics, because it is possible to detect possible epidemiological connections between the spread of one strain across the clinic during an invasive examination procedure, such as endoscopy [19,20].

## 2. Materials and Methods

### 2.1. Study Design and Setting, Inclusion Criteria

A prospective cohort study of midstream urine samples from patients with suspected lower urinary tract infection between January and October 2024 was carried out. The study included patients with clinical signs of lower UTI over the age of 18. The patients were from two different types of medical facilities, the Urocentrum (UC) centre of comprehensive uro-oncological care, an outpatient clinic focused on diagnosis and treatment of urological, oncological and uro-oncological diseases, and from the Urology Clinic of the General University Hospital (GUH) in Prague, which also provides comprehensive health care, including oncology with the possibility of hospitalization. The causative agent was identified in all samples (MALDI-TOF, Bruker Daltonik, Bremen, Germany) and susceptibility to selected antibiotics was determined according to EUCAST. A disc diffusion method was routinely performed, with an interpretation of the result as “sensitive/resistant”. The results were recorded in the Janiga Laboratory Information System (LIS). For subsequent analysis and creation of cumulative antibiograms, the data were extracted from LIS into Excel files. The data included information on the causative agent of the infection, antibiogram, information on age (years), gender, diagnosis, and medical facility.

The aim of the study was to analyze and compare culture findings and antibiograms based on primary diagnosis (lower UTI) in patients with complicated acute cystitis (cAC) and in patients with cAC in the field of pre-existing prostate carcinoma (N30 and C61 by International Classification of Diseases, 10th Revision diagnosis codes), according to the patient’s age and type of medical facility, focusing on the most isolated causative agent of *E. coli*.

Given that the patients showed symptoms of cAC, where urine culture is the standard of care, Ethics Committee approval was not required. For the purposes of the study, the data were completely anonymized.

### 2.2. Statistics

Where relevant, patient’s data were stratified according to age, gender, diagnosis and health care facility. Values are expressed as the number of cases and percentages from a group. The chi-square test was utilized to compare differences between diagnosis groups and/or age categories for particular substances. *p* < 0.05 was considered statistically significant. Statistical analyses were performed with MedCalc^®^ Statistical Software, version 23.0.2 (MedCalc Software Ltd., Ostend, Belgium; https://www.medcalc.org; accessed on 13 November 2024).

## 3. Results

### 3.1. Patient’s Population and Culture Findings

In the monitored period from January to October 2024, 383 patients from the Department of Urology of GUH were examined, of which 313 patients were diagnosed with N30, and 70 patients with C61, and 272 patients from the UC, of which 134 were diagnosed with N30, and 138 with C61.

The most common causative agent was *E. coli*, but the frequency of occurrence was much higher in patients with cAC; 261 (70%) isolates were found in GUH, and 69 (54%) isolates were found in UC, compared to 40 (39%) and 47 (35%), respectively, in patients with prostate cancer (Table 1). At the same time, the detection of other Gram-negative rods from the Enterobacterales family (*Proteus* sp., *Klebsiella* sp., *Morganella* and others) was higher in the cohort of oncology patients; in patients from the UC, they even outnumbered the incidence of *E. coli*, with 44% vs. 35% (Table 2).

### 3.2. Sub-Analysis of Antibiotic Susceptibility in Escherichia coli Isolates According to Diagnosis for the Same Medical Facility

**Figure 1:**
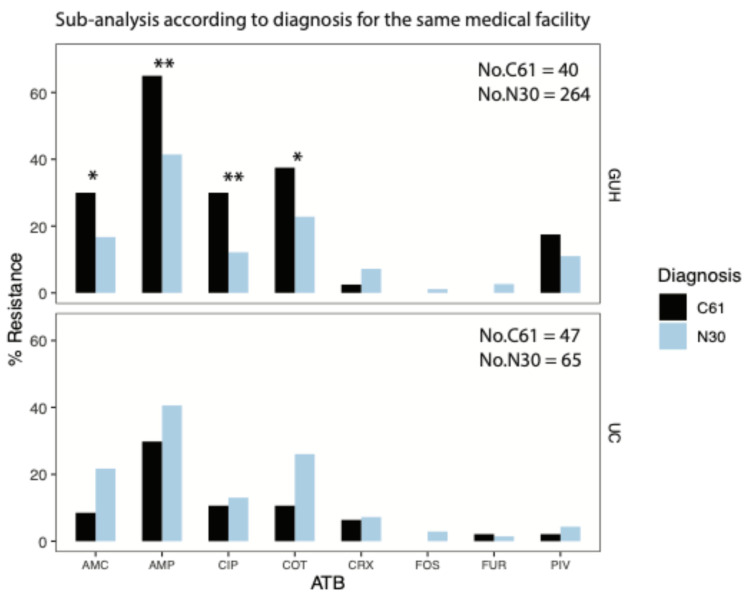
Antibiotic susceptibility of *Escherichia coli* isolates according to diagnosis for the same medical facility. Abbreviations: GUH—General University Hospital; UC—Urocentrum; AMP—ampicillin; AMC—amoxicillin/clavulanic acid; CRX—cefuroxime; PIV—pivmecillinam; COT—Co-trimoxazole; CIP—ciprofloxacin; FUR—nitrofurantoin; FOS—phosphomycin; N30—patients with complicated acute cystitis; C61—patients complicated acute cystitis and prostate cancer. * *p* < 0.05, ** *p* < 0.01.

A sub-analysis of the resistance profile of *E. coli* isolates related to the diagnosis showed significant differences between isolates from patients with N30 and those with C61 (Figure 1). *E. coli* isolates from patients with C61 were significantly more resistant to penicillins, including potentiated ones, in GUH patients (ampicillin, amoxicillin/c. clavulanic), co-trimoxazole and fluoroquinolones than *E. coli* isolates from patients with N30; 41% vs. 65% (*p* = 0.005), 17% vs. 30% (*p* = 0.04); 23% vs. 38% (*p* = 0.045); and 12% vs. 30% (*p* = 0.003), respectively. The opposite trend was recorded in patients from the UC, where *E. coli* isolates fromN30 were more resistant, albeit not statistically significantly, to penicillins, including potentiated ones, in GUH patients (ampicillin, amoxicillin/c. clavulanic), with co-trimoxazole and fluoroquinolones, than *E. coli* isolates from patients with C61, at 41% vs. 30% (*p* = 0.27), 22% vs. 9% (*p* = 0.07), 26% vs. 11% (*p* = 0.06), and 13% vs. 11% (*p* = 0.87), respectively. The difference is alarming and clinically significant in potentiated aminopenicillin and co-trimoxazole, where resistance is 2 to 2.5 times higher in N30 than in C61.

### 3.3. Sub-Analysis of Antibiotic Susceptibility in Escherichia coli Isolates by Medical Facility for the Same Diagnosis

**Figure 2:**
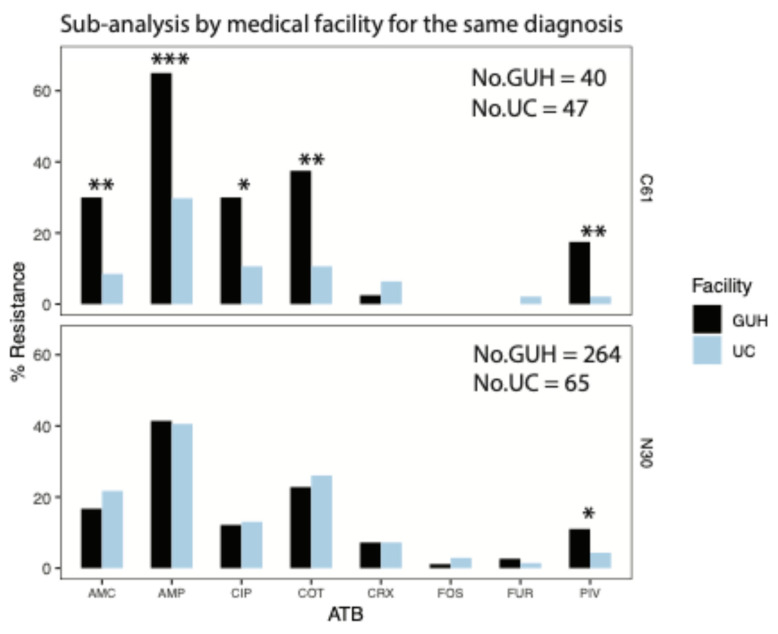
Antibiotic susceptibility of *Escherichia coli* isolates by medical facility for the same diagnosis. Abbreviations: GUH—General University Hospital; UC—Urocentrum; AMP—ampicillin; AMC—amoxicillin/clavulanic acid; CRX—cefuroxime; PIV—pivmecillinam; COT—Co-trimoxazole; CIP—ciprofloxacin; FUR—nitrofurantoin; FOS—phosphomycin; N30—patients with complicated acute cystitis; C61—patients complicated acute cystitis and prostate cancer. * *p* < 0.05, ** *p* < 0.01, *** *p* < 0.001.

When comparing the results of susceptibility testing of urinary tract isolates from patients with N30 between two different medical facilities, the results are comparable, except in the case of pivmecillinam, where resistance from patients from the outpatient UC is 4% and from GUH is 11% (*p* = 0.05) (Figure 2). However, the resistance profile is completely different in *E. coli* isolates from patients with C61 UTIs between the two different medical facilities. Isolates from UC were significantly more susceptible to ampicillin, amoxicillin/c. clavulanic, pivmecilinam, co-trimoxazole and fluorinated quinolones than isolates from GUH, with 31% vs. 65% (*p* = 0.001), 9% vs. 30% (*p* = 0.01), 2% vs. 18% (*p* = 0.001), 11% vs. 38% (*p* = 0.003) and 11% vs. 30% (*p* = 0.049), respectively. The level of resistance in GUH is at least twice as high, up to nine times in the case of pivmecilinam, which is alarming.

### 3.4. Age-Specific Sub-Analysis of Antibiotic Susceptibility in Escherichia coli Isolates

**Figure 3:**
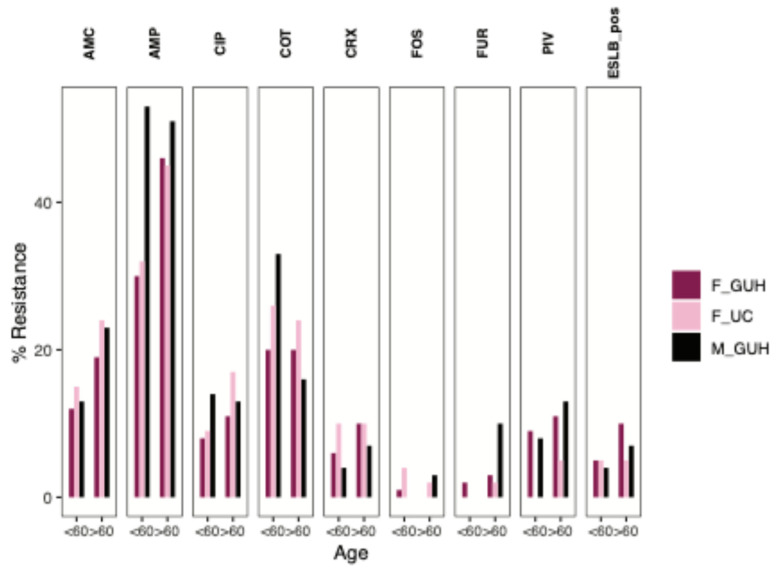
Comparison of *Escherichia coli* isolates’ resistances in N30 by gender, age, and health care facility. Abbreviations: AMP—ampicillin; AMC—amoxicillin/clavulanic acid; CRX—cefuroxime; PIV—pivmecillinam; COT—Co-trimoxazole; CIP—ciprofloxacin; FUR—nitrofurantoin; FOS—phosphomycin; ESLB—extended spectrum of betalactamases; F—female; M—male; GUH—General University Hospital; UC—Urocentrum.

The sub-analysis of *E. coli* isolates showed differences not only when comparing diagnoses and types of health care facilities, but also between age categories (Figure 3). For the age sub-analysis, we selected *E. coli* isolates from patients with N30 due to sufficient data. Isolates from both men (66 strains) and women (196 strains) were evaluated from GUH, and only isolates from women (66 strains) were evaluated from UC, because there were not enough men for analysis. The levels of resistance in isolates from patients younger and older than 60 years of age were evaluated. *E. coli* isolates from women over 60 years of age compared to isolates from the age category of under 60 years of age from both health care facilities had a higher level of resistance in all monitored antibiotics except co-trimoxazole and phosphomycin. In isolates from men, higher levels of resistance were reported for potentiated aminopenicillin, cefuroxime, pivmecillinam, nitrofurantoin, and phosphomycin. On the other hand, co-trimoxazole had a 2-fold lower resistance in the age category over 60 years, at 33% vs. 16% (Figure 3). *E. coli* isolates from GUH showed a consistently higher frequency of broad-spectrum beta-lactamase-producing strains in the age category over 60 years, with 4% vs. 7% in men and 5% vs. 10% in women. In UC isolates, the incidence of broad-spectrum beta-lactamase producers was the same in both age categories, namely 5%.

### 3.5. Sub-Analysis of the Incidence of Susceptible and Multidrug-Resistant Strains of Escherichia coli by Age, Diagnosis, Gender, and Health Care Facility

In individual patient cohorts, we analyzed the incidence of *E. coli* strains with different resistance phenotypes, ranging from completely susceptible to all antibiotics, through resistance to one, two, or more antibiotics, and including the occurrence of MDR strains, according to the definition [21].

**Figure 4:**
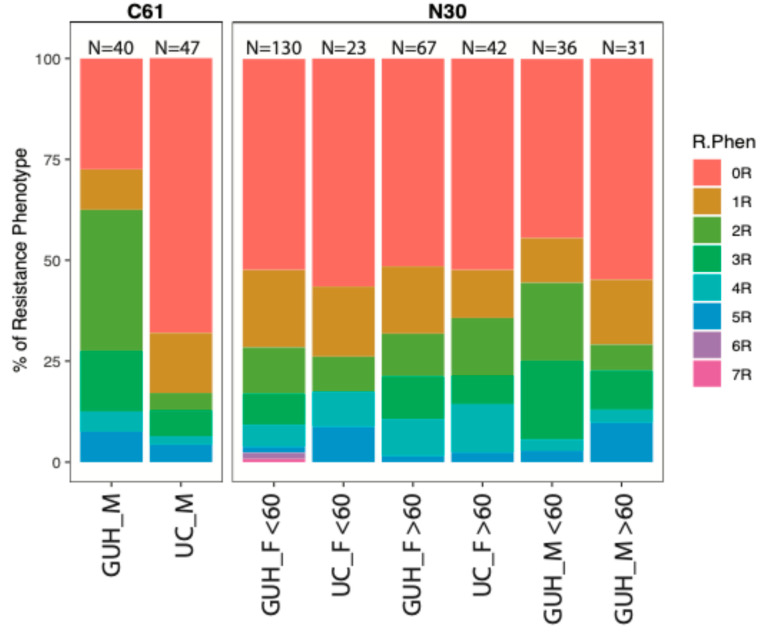
Incidence of susceptible and multidrug-resistant strains of *Escherichia coli* by age, diagnosis, gender, and health care facility. Abbreviations: GUH—General University Hospital; UC—Urocentrum; N30—patients with complicated acute cystitis; C61—patients complicated acute cystitis and prostate cancer, F—female; M—male; R.Phen—resistance phenotype; 0R—fully susceptible strain; 1R—resistant to one antibiotic; 2R–7R—number of resistant antibiotics.

There was a striking difference in patients diagnosed with C61 between the two centres, where the detection of fully sensitive isolates was only 27.5% in GUH and 68.1% in UC (Figure 4). A total of 65% of strains in GUH were resistant to aminopenicillins compared to 30% in UC, detection of MDR strains was 15% and 12.8%, respectively. In women diagnosed with N30, the proportion of fully sensitive strains was more than 50% in both health care facilities and age groups; however, in GUH, the incidence of MDR strains in the age category over 60 years was only 9%, compared to UC, where it was 16.7%; in those under 60 years old, it was only 7.7% versus 17.4%, i.e., more than double the incidence of MDR strains in an outpatient facility.

Fortunately, no isolate met the criteria of DTR (difficult to treat) [22]. All isolates were susceptible to carbapenems and amikacin, but these are only available in parenteral form, and, therefore, they are not applicable in the management of lower UTIs in the outpatient regimen in the Czech Republic.

## 4. Discussion

The implementation of cumulative antibiograms in clinical decision-making tools—such as guidelines and local information systems—is crucial for improving the accuracy of antibiotic prescribing practises, and it is one of the factors contributing to the reduction of overuse and/or inappropriate use of antibiotics in the treatment of the most common bacterial infections [23]. The most common cause of UTIs in children and adults, both in the community and in hospitals, is *E. coli* [24]. This fact might lead to assumptions that antibiotic therapy of UTIs, especially the uncomplicated ones, is relatively easy.

In our cohort, *E. coli* was the dominant causative agent in patients with N30 in GUH (70%), but in the UC, *E. coli* accounted only for 54% of cases. Among prostate cancer patients, the frequency of *E. coli* was markedly lower, at 39% in GUH and at 35% in UC. This is a clinically significant difference in the etiology of lower UTIs in relation to diagnosis compared to what has been seen in other published data [25,26]. Due to the high proportion of other bacteria in the etiology of UTIs in patients with comorbidities, this aspect should be included in the decision-making process for the choice of initial antibiotic. Based on the clinical condition of the patient, it is hard to identify both the causative agent and whether the causative agent is sensitive to antibiotics. The severity of the symptoms is determined by virulence factors, not antibiotic resistance [27].

The growing resistance of *E. coli* strains isolated from patients with UTIs is alarming. The resistance levels to commonly prescribed antibiotics are gradually narrowing treatment options for lower UTIs and patients often need to be hospitalized for parenteral antibiotic administration [28]. In the Czech Republic, there are national guidelines for the treatment of UTIs in adult patients [29], but all national guidelines are based on national epidemiological data [30]. However, this may not always reflect the situation at different health care facility levels as these guidelines do not consider other factors such as the patient’s age and possible comorbidities, or the type of health care facility presiding.

For the above reasons, a range of sub-analyses should be performed if enough different isolates are tested to ensure the statistical validity of the resistance estimates of individual subgroups, including stratification of isolates. The data we presented are unique not only in the Czech Republic, but also in other countries. Most published data compare regional differences, differences in age categories, gender-stratified data, or differences between community-acquired infections and nosocomial infections [31,32]. But, sub-analyses of data related to diagnosis, comorbidities, and presumed origin of infection are unique, and shows significant differences between antibiotics in relation to both diagnosis and origin of infection. Significant differences were observed between two health care facilities in patients with comorbidities, being, in our case, those with prostate cancer. Not only clinically significant, but also statistically significant differences were recorded for ampicillin, amoxicillin/clavulanic acid, cotrimoxazole, and ciprofloxacin in GUH between N30 and C61 patients who visit a health care facility more often for their primary diagnosis and who are exposed to certain epidemiological pressures. These findings are consistent with data from other studies where the level of resistance is higher in isolates from hospital settings [31]. However, the same analysis of data from the UC showed an opposite trend, where isolates from patients with prostate cancer showed clinically significantly lower resistance than isolates from patients with N30 to the same antibiotics. When comparing data from patients with the same comorbidity, but in different health care facilities, the difference in resistance to basic antibiotics is even more alarming, yet not always significant. For practical use, the use of statistical significance in the form of determining *p* value is slightly misleading and is influenced by the number of test subjects. The difference in the level of resistance expressed in percentage terms is more suitable for real practice and shows clinically significant differences in resistance that need to be used in routine practice.

We believe that the lower resistance in C61 patients from the UC might be explained by a lower antibiotic pressure in an outpatient facility. However, this does not explain why isolates from C61 patients are less resistant than isolates from N30 patients in the same outpatient facility. Hence, further investigation and more data are needed to validate this observation.

Evaluation of the sensitivity of individual antibiotics is important for each individual patient and for adjustment of the therapy used. If the antibiogram is not known at the time of choosing the initial treatment, it is more appropriate to monitor the occurrence of associated resistance, or to monitor the proportion of jointly sensitive antibiotics, especially in patients with a more severe course of infections, where there is no time to wait for the results of the culture examination and the confirmation of resistance [33,34,35]. In our study, there was a striking difference in the incidence of fully sensitive isolates in patients with oncological diagnosis (prostate cancer), where fully sensitive isolates only made up 27.5% in GUH, and 68% in UC, while the proportion of MDR strains was comparable and insignificant in both groups. On the other hand, the incidence of MDR isolates in women diagnosed with N30 is lower in GUH irrespective of age, relative to in an outpatient facility. Yet, the overall level of MDR isolates is higher compared to both national and international data [30,36]. Unfortunately, the only available comparable data are not from the current period, where a higher level of resistance in bacterial pathogens across disciplines or diagnoses is generally observed after the COVID-19 era [37].

There are several factors involved in the emergence and development of bacterial resistance to antibiotics, but the consumption of antibiotics plays an undeniable role [38,39]. A possible explanation for the enormous increase in the resistance of *E. coli* to protected penicillins in our data might be due to an increase in the consumption of protected penicillins in the Czech Republic, where, in 2008, the consumption was 3.7 defined daily doses (DDD)/1000 inhabitants/day, and in 2018, was 5 DDD/1000 inhabitants/day. When compared to some other European countries, the increase is up to five-fold; for example, in Norway in 2018, the consumption of protected penicillins was only 0.1 DDD/1000 inhabitants/day, and in Germany was 0.58 DDD/1000 inhabitants/day [40].

In this pilot study, we wanted to highlight the importance of stratified cumulative antibiograms at the level of individual health care facilities. The main limitations of the work include the lack of male patients diagnosed with N30 in an outpatient facility and the impossibility of comparing this cohort with patients from GUH. However, the data obtained are so serious and alarming that we will continue to collect and analyze the possible causes of such diametrically opposed results between two health facilities in the same city and to adjust the national guidelines for the treatment of urinary tract infections.

## 5. Conclusions

The most common causative agent in N30 was *Escherichia coli*. However, there was a high proportion of other Enterobacterales in C61. This fact should be considered when choosing an initial therapy, and laboratories should provide regular reports on the most common causative agents and their resistance profiles. Regularly generated cumulative antibiograms show trends in the development of bacterial resistance, which makes it possible to define potential problems for antimicrobial stewardship. *E. coli* isolates from patients with C61 were significantly more resistant to penicillins, including potentiated ones, in GUH than *E. coli* isolates from patients with N30. Furthermore, we have shown that resistance profiles of isolates from patients with the same diagnosis differed between our two medical facilities, highlighting the importance of stratification and sub-analysis of data according to defined parameters for the purposes of cumulative antibiograms.

## Figures and Tables

**Table 1:** Characteristics of patients with *E. coli* infection.

	GUH		UC	
	N30	C61	N30	C61
**Number of patients**	261	40	69	47
**gender**				
**Male**	67	40	4	47
**Female**	194	0	65	0
**Age, median years (IQR)**	48 (30–71)	78 (71–87.7)	66 (54.5–76)	77 (74–80)

Abbreviations: GUH—General University Hospital; UC—Urocentrum; N30—patients with complicated acute cystitis; C61—patients complicated acute cystitis and prostate cancer.

**Table 2:** Representation of culture findings according to diagnosis and health facility.

	C61		N30	
	GUH (%)	UC (%)	GUH (%)	UC (%)
***E. coli***	39	35	70	54
**Enterococci**	18	18	6	24
**other Enterobacterales**	28	44	19	14
**Nonfermenting rods**	12	3	2	0
**other G+ cocci**	0	0	3	6
***Candida* sp.**	3	0	0	2

Abbreviations: GUH—General University Hospital; UC—Urocentrum; N30—patients with complicated acute cystitis; C61—patients complicated acute cystitis and prostate cancer.

## Data Availability

The data used for the study are included in the article. Further inquiries can be directed to the corresponding authors.

## References

[1] Flores-Mireles A.L., Walker J.N., Caparon M., Hultgren S.J. (2015). Urinary tract infections: Epidemiology, mechanisms of infection and treatment options. Nat. Rev. Microbiol..

[2] Medina M., Castillo-Pino E. (2019). An introduction to the epidemiology and burden of urinary tract infections. Ther. Adv. Urol..

[3] Foxman B., Barlow R., D’Arcy H., Gillespie B., Sobel J.D. (2000). Urinary tract infection: Self-reported incidence and associated costs. Ann. Epidemiol..

[4] Zhu N.J., Weldegiorgis M., Carter E., Brown C., Holmes A., Aylin P. (2024). Economic Burden of Community-Acquired Antibiotic-Resistant Urinary Tract Infections: Systematic Review and Meta-Analysis. JMIR Public Health Surveill..

[5] Gupta K., Grigoryan L., Trautner B. (2017). Urinary Tract Infection. Ann. Intern. Med..

[6] Kranz J., Bartoletti R., Bruyère F., Cai T., Geerlings S., Köves B., Schubert S., Pilatz A., Veeratterapillay R., Wagenlehner F.M.E. (2024). European Association of Urology Guidelines on Urological Infections: Summary of the 2024 Guidelines. Eur. Urol..

[7] Huang L., Huang C., Yan Y., Sun L., Li H. (2022). Urinary Tract Infection Etiological Profiles and Antibiotic Resistance Patterns Varied Among Different Age Categories: A Retrospective Study From a Tertiary General Hospital During a 12-Year Period. Front. Microbiol..

[8] Bader M.S., Loeb M., Brooks A.A. (2017). An update on the management of urinary tract infections in the era of antimicrobial resistance. Postgrad. Med..

[9] Hrbacek J., Cermak P., Zachoval R. (2020). Current Antibiotic Resistance Trends of Uropathogens in Central Europe: Survey from a Tertiary Hospital Urology Department 2011–2019. Antibiotics.

[10] Aronin S.I., Gupta V., Dunne M.W., Watts J.A., Yu K.C. (2022). Regional Differences in Antibiotic-resistant Enterobacterales Urine Isolates in the United States: 2018–2020. Int. J. Infect. Dis..

[11] Spoorenberg V., Hulscher M.E., Akkermans R.P., Prins J.M., Geerlings S.E. (2014). Appropriate antibiotic use for patients with urinary tract infections reduces length of hospital stay. Clin. Infect. Dis..

[12] Critchley I.A., Karlowsky J.A. (2004). Optimal use of antibiotic resistance surveillance systems. Clin. Microbiol. Infect..

[13] World Health Organisation (WHO) (2007). Global action plan on antimicrobial resistance. Geneva: WHO; 2015. Available from Hindler JF, Stelling J. Analysis and presentation of cumulative antibiograms: A new consensus guideline from the Clinical and Laboratory Standards Institute. Clin. Infect. Dis..

[14] Eden C., Ackermann F. (2013). Making Strategy: The Journey of Strategic Management.

[15] CLSI (2014). Analysis and Presentation of Cumulative Antimicrobial Susceptibility Test Data.

[16] Moehring R.W., Hazen K.C., Hawkins M.R., Drew R.H., Sexton D.J., Anderson D.J. (2015). Challenges in Preparation of Cumulative Antibiogram Reports for Community Hospitals. J. Clin. Microbiol..

[17] Laupland K.B., Ross T., Pitout J.D., Church D.L., Gregson D.B. (2007). Investigation of sources of potential bias in laboratory surveillance for anti-microbial resistance. Clin. Investig. Med..

[18] Klinker K.P., Hidayat L.K., DeRyke C.A., DePestel D.D., Motyl M., Bauer K.A. (2021). Antimicrobial stewardship and antibiograms: Importance of moving beyond traditional antibiograms. Ther. Adv. Infect. Dis..

[19] https://www.who.int/publications/i/item/9789241509763.

[20] Simner P.J., Hindler J.A., Bhowmick T., Das S., Johnson J.K., Lubers B.V., Redell M.A., Stelling J., Erdman S.M. (2022). What’s New in Antibiograms? Updating CLSI M39 Guidance with Current Trends. J. Clin. Microbiol..

[21] Magiorakos A.P., Srinivasan A., Carey R.B., Carmeli Y., Falagas M.E., Giske C.G., Harbarth S., Hindler J.F., Kahlmeter G., Olsson-Liljequist B. (2012). Multidrug-resistant, extensively drug-resistant and pandrug-resistant bacteria: An international expert proposal for interim standard definitions for acquired resistance. Clin. Microbiol. Infect..

[22] Kadri S.S., Adjemian J., Lai Y.L., Spaulding A.B., Ricotta E., Prevots D.R., Palmore T.N., Rhee C., Klompas M., Dekker J.P. (2018). Difficult-to-Treat Resistance in Gram-negative Bacteremia at 173 US Hospitals: Retrospective Cohort Analysis of Prevalence, Predictors, and Outcome of Resistance to All First-line Agents. Clin. Infect. Dis..

[23] Neugebauer M., Ebert M., Vogelmann R. (2020). A clinical decision support system improves antibiotic therapy for upper urinary tract infection in a randomized single-blinded study. BMC Health Serv. Res..

[24] Schaeffer A., Schaeffer E., Wein A., Kavoussi L. (2012). Infections of the urinary tract. Campbell-Walsh Urology.

[25] Toval F., Köhler C.-D., Vogel U., Wagenlehner F., Mellmann A., Fruth A., Schmidt M.A., Karch H., Bielaszewska M., Dobrindt U. (2014). Characterization of *Escherichia coli* isolates from hospital inpatients or outpatients with urinary tract infection. J. Clin. Microbiol..

[26] Herbawi A., Abu Taha A., Aiesh B.M., Sabateen A., Zyoud S.H. (2024). Spectrum and antibiotic resistance in community- and hospital-acquired urinary tract infections among adults: Experience from a large tertiary care center in a developing country. Urologia.

[27] Cepas V., Soto S.M. (2020). Relationship between Virulence and Resistance among Gram-Negative Bacteria. Antibiotics.

[28] Critchley I.A., Cotroneo N., Pucci M.J., Mendes R. (2019). The burden of antimicrobial resistance among urinary tract isolates of Escherichia coli in the United States in 2017. PLoS ONE.

[29] Matoušková M., Adámková V., Čechová M., Lahoda Brodská H. (2022). Konsenzuální Postupy v Léčbě Močových Infekcí.

[30] https://szu.gov.cz/wp-content/uploads/2023/12/PSMR_2021_EC.pdf.

[31] Corse L., Cartwright A. (2024). Investigating trends in antibiotic resistance of *Escherichia coli* isolated from clinical urine specimens in the Orkney Islands. Microbiology.

[32] Jorgensen S., Zurayk M., Yeung S., Terry J., Dunn M., Nieberg P., Wong-Beringer A. (2017). Emergency Department Urinary Antibiograms Differ by Specific Patient Group. J. Clin. Microbiol..

[33] Goodlet K.J., Nicolau D.P., Nailor M.D. (2017). In Vitro Comparison of Ceftolozane-Tazobactam to Traditional Beta-Lactams and Ceftolozane-Tazobactam as an Alternative to Combination Antimicrobial Therapy for *Pseudomonas aeruginosa*. Antimicrob. Agents Chemother..

[34] Moise P.A., Gonzalez M., Alekseeva I., Lopez D., Akrich B., DeRyke C.A., Chen W.T., Pavia J., Palermo B., Hackel M. (2021). Collective assessment of antimicrobial susceptibility among the most common Gram-negative respiratory pathogens driving therapy in the ICU. JAC Antimicrob. Resist..

[35] Wangchinda W., Aitken S.L., Klatt M.E., Lephart P.R., Smith A.B., Pogue J.M. (2024). A Comparison of Different Strategies for Optimizing the Selection of Empiric Antibiotic Therapy for Pneumonia Caused by Gram-Negative Bacteria in Intensive Care Units: Unit-Specific Combination Antibiograms Versus Patient-Specific Risk Factors. Open Forum Infect. Dis..

[36] Kaye K.S., Gupta V., Mulgirigama A., Joshi A.V., Scangarella-Oman N.E., Yu K., Ye G., Mitrani-Gold F.S. (2021). Antimicrobial Resistance Trends in Urine *Escherichia coli* Isolates From Adult and Adolescent Females in the United States From 2011 to 2019: Rising ESBL Strains and Impact on Patient Management. Clin. Infect. Dis..

[37] Golli A.-L., Zlatian O.M., Cara M.L., Olteanu M. (2024). Pre- and Post-COVID-19 Antimicrobial Resistance Pattern of Pathogens in an Intensive Care Unit. Pharmaceuticals.

[38] Stapleton P.J., Lundon D.J., McWade R., Scanlon N., Hannan M.M., O’Kelly F., Lynch M. (2017). Antibiotic resistance patterns of *Escherichia coli* urinary isolates and comparison with antibiotic consumption data over 10 years, 2005–2014. Ir. J. Med. Sci..

[39] Holm A., Cordoba G., Aabenhus R. (2019). Prescription of antibiotics for urinary tract infection in general practice in Denmark. Scand. J. Prim. Health Care.

[40] Central Coordination Group of the National End of the Czech Republic: Antibiotic consumption in the Czech Republic in 2008–2018—Part 1. Pharmacotherapeutic Information 1/2020. http://www.sukl.cz/sukl/2020.

